# Crystallization of spin superlattices with pressure and field in the layered magnet SrCu_2_(BO_3_)_2_

**DOI:** 10.1038/ncomms11956

**Published:** 2016-06-20

**Authors:** S. Haravifard, D. Graf, A. E. Feiguin, C. D. Batista, J. C. Lang, D. M. Silevitch, G. Srajer, B. D. Gaulin, H. A. Dabkowska, T. F. Rosenbaum

**Affiliations:** 1 Department of Physics, Duke University, Durham, North Carolina 27708, USA; 2 The James Franck Institute and Department of Physics, The University of Chicago, Chicago, Illinois 60637, USA; 3 Advanced Photon Source, Argonne National Laboratory, Argonne, Illinois 60439, USA; 4 National High Magnetic Field Laboratory and Department of Physics, Florida State University, Tallahassee, Florida 32310, USA; 5 Department of Physics, Northeastern University, Boston, Massachusetts 02115, USA; 6 Department of Physics, University of Tennessee, Knoxville, Tennessee 37996, USA; 7 Quantum Condensed Matter Division and Shull-Wollan Center, Oak Ridge National Laboratory, Oak Ridge, Tennessee 37831, USA; 8 Theoretical Division, Los Alamos National Laboratory, Los Alamos, New Mexico 87545, USA; 9 Division of Physics, Mathematics & Astronomy, California Institute of Technology, Pasadena, California 91125, USA; 10 Department of Physics and Astronomy and Brockhouse Institute for Material Research, McMaster University, Hamilton, Ontario, Canada L8S 4M1

## Abstract

An exact mapping between quantum spins and boson gases provides fresh approaches to the creation of quantum condensates and crystals. Here we report on magnetization measurements on the dimerized quantum magnet SrCu_2_(BO_3_)_2_ at cryogenic temperatures and through a quantum-phase transition that demonstrate the emergence of fractionally filled bosonic crystals in mesoscopic patterns, specified by a sequence of magnetization plateaus. We apply tens of Teslas of magnetic field to tune the density of bosons and gigapascals of hydrostatic pressure to regulate the underlying interactions. Simulations help parse the balance between energy and geometry in the emergent spin superlattices. The magnetic crystallites are the end result of a progression from a direct product of singlet states in each short dimer at zero field to preferred filling fractions of spin-triplet bosons in each dimer at large magnetic field, enriching the known possibilities for collective states in both quantum spin and atomic systems.

## Abstract (web summary)

Spin 1/2 dimer systems in an external magnetic field behave as a lattice gas of hard-core bosons, and can undergo condensation. Here the authors show in SrCu_2_(BO_3_)_2_ that new fractionally crystallized states are not only possible, but also tuneable with hydrostatic pressure.

## Graphical abstract

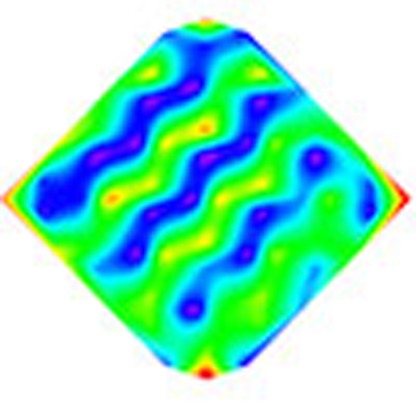

The condensation of a gas of identical, non-interacting bosons—fundamental particles with integer spin—into the lowest energy level represents a canonical demonstration of emergent quantum behaviour. This phenomenon has been observed in a wide range of physical systems, ranging from superfluidity in helium-4^1^ to dilute gases of cold atoms^2^, and is now known as Bose–Einstein condensation (BEC) after the pioneering quantum statistical predictions of 1924. BEC effects also can be induced in fermionic materials such as helium-3, where particles of half-integer spin form bound pair states, which then condense at low temperatures^3^,^4^. Surprisingly, quantum magnets can fulfil this prescription, where an analogy is drawn between a spin 1/2 dimer system in an external magnetic field *H* and a lattice gas of hard-core bosons^5^,^6^,^7^,^8^. We show here that it is not only possible to crystallize the quantum spins of the model two-dimensional (2D) magnet SrCu_2_(BO_3_)_2_, composed of sheets of Cu^2+^ spin 1/2 dimers on a square lattice, into mesoscopic bosonic patterns under the influence of large magnetic fields, but also to tune these patterns with hydrostatic pressure.

At *H=*0, pairs of antiferromagnetically coupled *S=*1/2 spins can dimerize to form effective spins with an *S=*0 ground state and an *S=*1 excited state separated by an energy gap Δ. When an external magnetic field is applied, the Zeeman effect breaks the degeneracy of the triplet state and suppresses the energy gap between the ground state and the lower branch of the excited state at a critical field *H*_c_*=*Δ/*gμ*_B_, where *g* is the electron *g*-factor and *μ*_B_ is the Bohr magneton (Fig. 1a). Dimers in the singlet ground state map to an unoccupied lattice site, while those in the excited triplet state correspond to a boson occupying a lattice site. Above *H*_c_, the gap is closed and a finite density of bosons can be sustained even at zero temperature, thus enabling the possibility of a BEC.

Physical realizations of dimerized spin systems typically have both inter- and intra-dimer interactions. These interactions map to repulsive and hopping terms, respectively, in the bosonic picture, with the applied field acting as the chemical potential. Systems where the spin topology leads to magnetic frustration exhibit reduced kinetic energy, and the triplets will then minimize the repulsive interactions by crystallizing. The resulting Wigner crystal can be characterized by the fractional filling factor of bosons on a superlattice. Here we tune the magnetic field to favour different superlattice filling factors along with applying hydrostatic pressure to regulate the underlying dimer interactions in a 2D quantum magnet, including passage through a quantum-phase transition, demarcating singlet physics and stripe order. Numerical simulations confirm the identities of the different states that emerge with the shifting balance of inter- and intra-dimer exchange interactions. The internal structure, energetic stability and evolution of the different superlattice states with pressure and field illuminate the collective dynamics of highly frustrated quantum spin systems.

The primary observable, both in experiment and in numerical simulations, is the overall magnetization *M*, expressed in normalized units as *m ≡ M*/*M*_sat_ where *M*_sat_ is the saturation moment, corresponding to all spins aligned with the applied field. When crystallization occurs, a gap opens in the excitation spectrum, and the boson density remains unchanged until the system transitions into a new phase. The magnetization fraction *m* of the corresponding magnetization plateau directly yields the filling factor. The topology we study here is the Shastry–Sutherland model^9^, a planar array of *S=*1/2 spins coupled by a network of nearest-neighbour (NN), *J*, and next-nearest-neighbour (NNN) interactions, *J′*, (Fig. 1b) with Hamiltonian


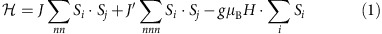


The ground-state spin configuration depends on the relative values of *J* and *J′*. For small *x ≡ J′/J*, the ground state is a collection of interacting singlet states on each dimer. There is a quantum-phase transition into an intermediate short-range-ordered state of antiferromagnetic plaquettes at *x*∼0.69, followed by a second transition into a Néel antiferromagnet at *x*∼0.8. The sensitivity of the dimer energy gap Δ on *x* makes the Shastry–Sutherland topology a valuable substrate for studying emergent bosonic superstructures, as it offers the ability to independently tune the interaction energy (via *x*) and the chemical potential (that is, the magnetic field *H*).

The layered spin-dimer material SrCu_2_(BO_3_)_2_ (SCBO) is an experimental realization of the Shastry–Sutherland model^10^,^11^,^12^,^13^, with *x* estimated to lie between 0.6 and 0.64 at ambient pressure^13^,^14^, and a measured singlet–triplet gap energy Δ=3 meV (ref. 15). The magnetization plateaus characteristic of bosonic crystallization emerge for *H*>20 T, first at filling fractions *m=*1/8, 1/4 and 1/3, and subsequently at intermediate fillings 2/15 and 1/6 for temperatures below ∼0.8 K (refs 6, 11, 16, 17, 18, 19). Recent magnetostriction measurements at fields up to 118 T have seen indications of *m=*2/5 and 1/2 plateaus^20^,^21^. Although clearly demonstrating bosonic crystallization in a quantum magnet, the exact spin configurations in the low-field plateau states remain a matter of debate^11^,^16^,^17^,^18^,^19^,^20^,^21^,^22^,^23^.

Tuning the other major variable in the problem, the dimer interaction ratio *x ≡ J′/J*, offers the possibility of further insights into the plethora of high-field collective states. Hydrostatic pressure tunes *x* across the phase diagram, with a continuous quantum-phase transition into the plaquette state at *P*∼2 GPa (refs 24, 25, 26, 27), followed by long-range antiferromagnetic order and an associated symmetry-breaking structural transition at *P∼*4.5 GPa (refs 28, 29). SCBO remains effectively 2D for pressures below this structural transition, at which point the Cu–Cu dimers tilt out of the plane^28^.

By varying *P* and sweeping *H*, we systematically study the bosonic crystal against a backdrop of different underlying spin states, controlling the strength of the kinetic and repulsive energies, as well as the overall chemical potential. To that end, we performed magnetization measurements on high-quality single crystals of SCBO in a piston cylinder cell for pressures up to 2.2 GPa, magnetic fields up to 34 T and temperatures down to 350 mK.

## Results

### High-field magnetization at ambient pressure

**Figure 1:**
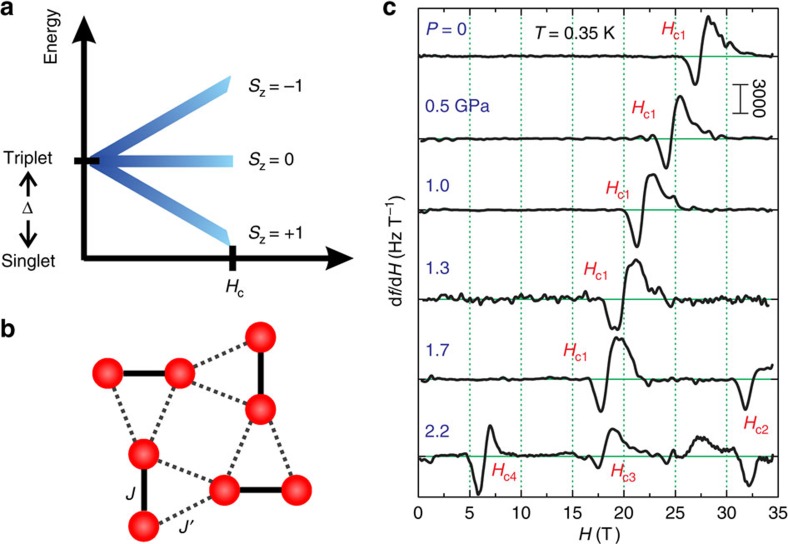
Pressure-driven tuning of magnetic crystal states in SrCu_2_(BO_3_)_2_. (**a**) Energy diagram of dimerized *S=*1/2 spins in a magnetic field. Zeeman splitting closes the zero-field singlet–triplet gap Δ at a critical field *H*_c_, allowing the formation of superlattices of triplets. (**b**) Spin topology of the Shastry–Sutherland model, as realised in magnetic layers of SrCu_2_(BO_3_)_2_. Red circles represent the *S*=1/2 Cu^2+^ ions. Dimerized spins (Cu^2+^ ions) are coupled together with nearest-neighbour interaction energy *J*; inter-dimer coupling mediated by next-nearest-neighbour interactions *J′*. (**c**) Tunnel diode oscillator measurements of the susceptibility d*f*/d*H* ∝ d*M*/d*H* of SrCu_2_(BO_3_)_2_, as a function of applied field at *T=*0.35 K for a series of pressures. A feature corresponding to the *m=*1/8 magnetization plateau (*H*_c1_) shifts continuously to lower field, as the pressure tunes the underlying interactions. Above 2 GPa, a quantum-phase transition results in the appearance of additional magnetic superlattice states at *H*_c2_, *H*_c3_ and *H*_c4_. A background measured at *T=*2 K has been subtracted from each curve. Curves shifted vertically for clarity.

Due to the small sample volumes available in the pressure cell, we employed a high-sensitivity tunnel diode oscillator (TDO) technique^30^ to measure the magnetization. The crystal is placed inside the inductor component of a diode-biased self-resonant LC tank circuit and changes in the sample magnetization lead to changes in the circuit resonant frequency *f* (Supplementary Figs 1,2). We plot in Fig. 1c the variation of the TDO resonant frequency with external magnetic field (d*f*/d*H*) at *T=*0.35 K for a range of pressures, where d*f*/d*H* ∝ d*M*/d*H*, the magnetic susceptibility of the sample. The magnetic field was applied parallel to the *c* axis of the crystal. At ambient pressure, an anomaly is clearly visible at *H*_c1_∼27 T that corresponds to the *m=*1/8 plateau observed in previous experiments^16^,^17^,^18^ and in our own *P=*0 torque magnetometry measurements (Supplementary Fig. 3), with no hysteresis detected between field up and down trajectories. The ∼2 T distance between the two extrema in the anomaly indicates the range of magnetic field at which the *m=*1/8 triplet superlattice phase exists. Nuclear magnetic resonance^17^,^19^ and magnetostriction^18^,^20^,^21^ measurements found that the superlattice states of highly magnetized triplet dimers persist adjacent to the 1/8 plateau for fields up to ∼30 T. These states may account for the skewed line shape of the maximum observed above *H*_c1_ in Fig. 1c.

### High-field magnetization at high pressure

The magnetic susceptibility responds sensitively to pressure, where the 2.2 GPa maximum *P* in Fig. 1c corresponds to ∼10% increase in *x,* with *P*_c_=1.93±0.07 GPa (refs 24, 31, 32). For *P*<*P*_c_, the continuous increase in *x* and consequent decrease in Δ drives the feature marking the *m=*1/8 superlattice plateau to lower field, with *H*_c1_ at only 2/3 of its *P=*0 value by 1.7 GPa. Crossing *P*_c_ changes the response not only quantitatively, but qualitatively as well. Instead of a single feature at *H*_c1_ corresponding to the *m=*1/8 filling, we observe a total of three features, which we denote as *H*_c2_ at 33 T, *H*_c3_ at 18 T and *H*_c4_ at 7 T. By pressure tuning the *H=*0 spin configuration into a different ground state, we enable a different set of high-field magnetic superlattice states to emerge.

### Temperature dependence of magnetization at different pressures

**Figure 2:**
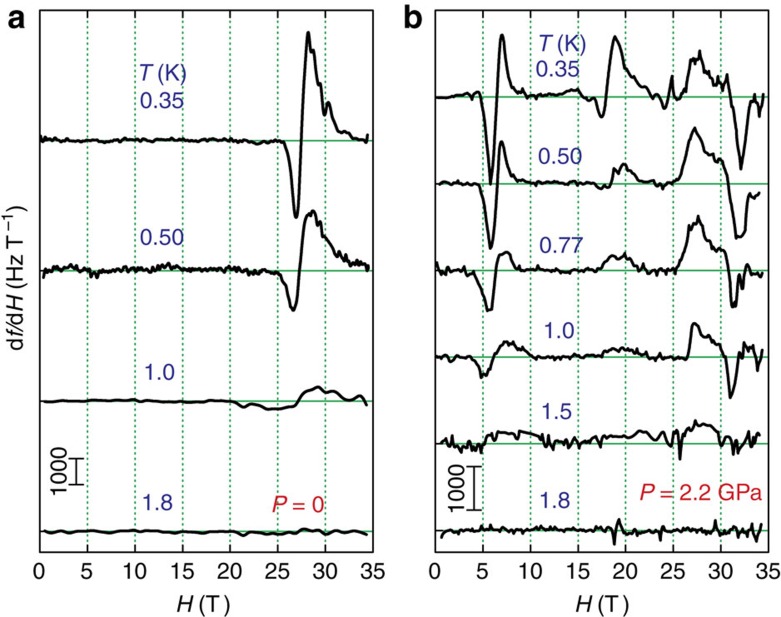
Temperature evolution of spin superlattice states in SrCu_2_(BO_3_)_2_. Temperature dependence of the variations in the resonant frequency of the TDO in response to magnetic field, obtained at *P=*0 (**a**) and *P=*2.2 GPa (**b**). In both regimes of pressures (**a**) below and (**b**) above the 2 GPa transition from singlet state to short-range-ordered plaquettes, the superlattice states melt with increasing temperature and vanish completely by 1.8 K. Traces shifted vertically for clarity; a background obtained at high temperature has been subtracted from all traces.

We now turn to the question of the energetics and microstructure of these new states. We plot in Fig. 2 the temperature dependence of the susceptibility both below and above *P*_c_. At ambient pressure, the signature of the *m=*1/8 plateau is suppressed with increasing *T* and is no longer detected at 1.8 K (Fig. 2a), comparable to prior measurements^11^,^17^,^18^. The new anomalies in the high-pressure state are also suppressed by *T=*1.8 K (Fig. 2b), where the spin superlattices of different *m* apparently melt. The associated values of the critical fields remain fixed over the entire temperature range, indicating that the amplitudes of the spin-density modulations are independent of *T*.

### Density matrix renormalization group calculation

To elucidate the spin structures in both the low *P*, singlet and the high *P*, plaquette states, we compare the experimental results with numerical calculations performed on an ideal Shastry–Sutherland lattice over a range of *x*. We calculate *M*(*H*) using a density matrix renormalization group (DMRG) approach on finite lattices of 8 × 8, 10 × 10 and 16 × 8 spins for *x ≡ J′/J≈*0.6, 0.63 and 0.7 with cylindrical boundary conditions (Supplementary Fig. 4). We found that this choice is less vulnerable to boundary effects, and is similar to the one employed in previous studies.^20^

**Figure 3:**
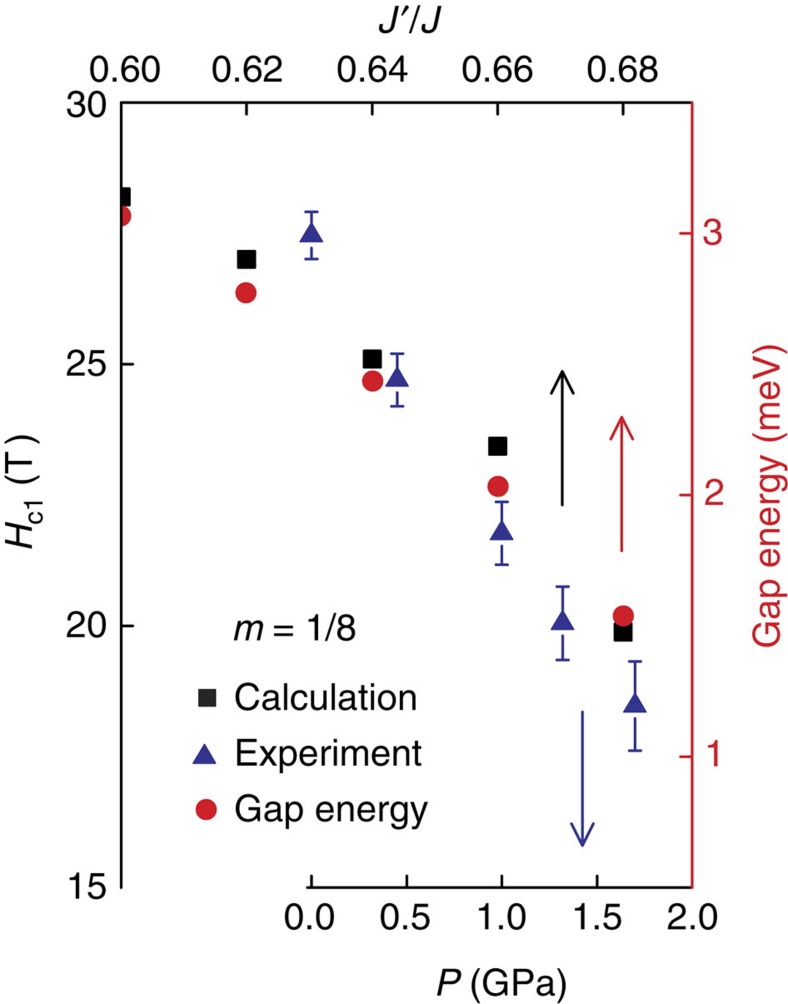
Evolution of the critical field of the *m*=1/8 magnetization plateau. (Black squares) DMRG simulations as a function of the interaction ratio *x=J′/J.* (Blue triangles) Experimental measurements on SrCu_2_(BO_3_)_2_ as a function of pressure. Error bars represent one standard error from mean (s.e.m.) calculated from fits to a thermally activated model^24^. Linear mapping between *x* and *P* is *x=*0.63 for *P=*0 and *x=*0.69 for *P=*2 GPa, determined from ambient pressure measurements of *x* and the critical pressure required to suppress the singlet–triplet gap and induce phase transition^24^,^25^,^26^,^27^,^31^,^32^. (Right axis) Red circles show the singlet–triplet gap energy calculated at zero field as a function of *x*.

We plot in Fig. 3, the calculated singlet–triplet gap Δ (red) and the critical field for the *m=*1/8 plateau (green), as a function of *x*=*J′/J* for an 8 × 8 lattice with cylindrical boundary conditions. We show the experimentally measured pressure evolution of *H*_c1_ (blue) on the lower axis, mapped to *x* on the upper axis^24^,^31^,^32^. *H*_c1_ for the *m*=1/8 plateau continuously decreases with increasing *P*, as the gap between the lowest triplet excitation branch and the singlet state decreases. The correspondence between the calculated and the measured values confirms the connection between *H*_c1_ and the suppression of the singlet–triplet gap Δ, and further, serves as a consistency check between the numerical results and the experimental data, with no free parameters in the comparison (Supplementary Fig. 5).

**Figure 4:**
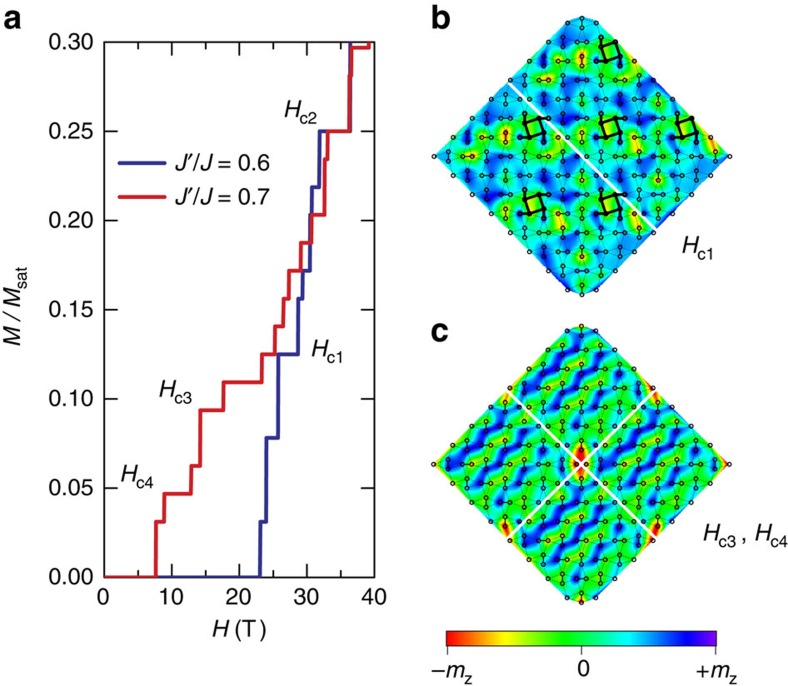
Simulations of magnetization profiles of SrCu_2_(BO_3_)_2_ as a function of inter- and intra-dimer interactions. (**a**) Magnetization curves obtained from DMRG calculations on a 16 × 8 spin lattice with cylindrical boundary conditions for the singlet phase (*J′/J=*0.6; blue) and the plaquette phase (*J′/J=*0.7; red). Features move to lower *H* and new plateaus emerge as *x* increases from 0.6 to 0.7, echoing the experimental results. (**b**,**c**) Schematics of the spin superlattice configuration corresponding to the *H*_c1_ and *H*_c3,c4_ plateaus mapped onto a natural realization of the Shastry–Sutherland lattice, and computed using 16 × 8 and 8 × 8 spin lattices with open boundary conditions, respectively (**b** consists of two 16 × 8 and **c** consists of four 8 × 8 spin lattices). Solid lines denote orthogonal dimers, and the colour bar represents the magnitude of the local magnetization, where +*m*_*z*_/−*m*_*z*_ indicate spins strongly polarized parallel/antiparallel to the field. The simulated superstructure opens the prospect of crystallization of ‘pinwheel' triplet bound states in a square lattice at *H*_c1_ and field-induced stripe ordering in the bound state of triplons at *H*_c3,c4_.

The calculated magnetization curves merge as a function of increased system size (Supplementary Fig. 4). We plot in Fig. 4a, the magnetization curves obtained using DMRG calculations for an 16 × 8 spin lattice with cylindrical boundary conditions for *x=*0.6 (blue) and 0.7 (red). These serve to represent the singlet and plaquette ground states, respectively^31^,^32^. New plateaus emerge for *x=*0.7, with a pronounced shift to lower *H*. While the resolution limits of the numerics are not sufficient to assign precise filling fractions to all of the plateaus seen in the calculation, the magnetization curve captures the essential features observed in the experimental data of Fig. 1c, including the emergence at *P=*2.2 GPa (*x=*0.7) of new anomalies at *H*_c4_∼7 T and *H*_c3_∼15 T. These results indicate that for *x=*0.7, the normalized magnetizations associated with *H*_c3_ and *H*_c4_ converge to filling factors of ∼1/10 and ∼1/20, both smaller than *m=*1/8, the lowest plateau seen for *x=*0.6 in the calculations and for *P<*2 GPa in the experiment.

## Discussion

To explore the nature of the new magnetization profile, we performed DMRG simulations at fixed magnetization *S*_*z*_ on lattices with different aspect ratios, keeping up to 3,000 DMRG states, corresponding to a truncation error of order 10^−5^ or smaller for the system sizes considered (Supplementary Fig. 6). We followed a zigzag path along the diagonal bonds of the square lattice, taking advantage of the strong entanglement along these links. The simulations are done on a conventional square lattice with open boundary conditions to avoid a bias towards magnetization profiles that break the 90° rotational symmetry of the lattice. We introduced a small pinning field at the site (*x*, *y*)=(*L*_*x*_, *L*_*y*_), where *L* is the linear dimension of the lattice, to resolve the spatial magnetization profile of the structure that has maximum susceptibility in a finite-size system, removing this field in the last series of iterations. For *x*=0.63 and a 16 × 8 lattice, we found a ground-state energy per site of *E*_0_=−0.3478 for *S*_*z*_=16 (corresponding to *m=*1/8). This value lies close to, but slightly above the extrapolation to the thermodynamic limit, *E*_0_=−0.3475 that was obtained by Corboz *et al.*^22^ using infinite projected entangled-pair states (iPEPS), a tensor-network method, for a structure formed by bound states of triplets. We note that the ground-state energy is expected to be higher for a finite system, due in part to the potential mismatch in aspect ratios between the finite lattice and the optimal magnetic structure.

We present in Fig. 4b,c schematics of the magnetization profiles, corresponding to the *H*_c1_ and *H*_c3,c4_ plateaus mapped onto a real-world realization of the Shastry–Sutherland lattice, and computed for 16 × 8 and 8 × 8 spin lattices with open boundary conditions along both directions, respectively. The magnetization profile at *H*_c1_, (Fig. 4b) is consistent with the iPEPS results^22^, and suggests the crystallization of *S*_*z*_=2 triplet bound states, which form a ‘pinwheel' pattern on a square supercell at the *m=*1/8 plateau. This agreement between the DMRG and iPEPS results is remarkable considering the unbiased nature of the DMRG approach. By contrast, the magnetization profiles obtained at *H*_c3_ and *H*_c4_ point to the likelihood of field-induced stripe ordering (Fig. 4c). The emergence of low-magnetization stripe-ordered magnetic superstructures indicates that the pressure-tuned increase in the kinetic energy of the triplets, eventually could lead to their full condensation into a long-range-ordered antiferromagnet. Stripe structures are also proposed to appear for higher-magnetization pleateaus, *m*=2/15, 1/6 and 1/4 at ambient pressure^22^. The full nature of these states will require further theoretical investigation, including the need to understand the role of spin–lattice coupling^33^ and anisotropic Dzyaloshinskii–Moriya interactions^28^,^34^.

The plateau at *H*_*c2*_ behaves differently than those at *H*_c3_ and *H*_c4_ in that it initially emerges in the experiment for *P<P*_*c*_. The *m=*1/4 state satisfies this criterion, being present in the simulations for both *x=*0.6 and 0.7. Filling factor 1/4 is believed to be especially stable^17^,^18^,^19^,^20^,^21^, and we posit that this superlattice state exists over the full range of the singlet and plaquette phases that we probe, moving into our experimental magnetic field window at 1.7 GPa and continuing to shift to lower *H* with increasing *P*. The stability of *H*_c2_ at *m=*1/4 contrasts with that of the *H*_c1_ plateau at *m=*1/8 that no longer persists across the quantum-phase transition at *P*_c_.

One of the long-standing mysteries of SCBO is the existence of bosonic crystals at *m* values as low as 1/8. As the Shastry–Sutherland model only has bare repulsive interactions between NN and NNN, low-density structures such as *m=*1/8, where the average distance between bosons is significantly larger than the distance between NN and NNN, are not expected to appear. Consequently, one can ask what is the origin of the longer length scale that determines the crystallization at *m*=1/8 and lower concentrations? One plausible explanation is given by the formation of triplet bound states. In this picture, the missing length scale is *l*_B_, the mean separation between the two *S*_*z*_=2 triplets in the bound state (analogous to the coherence length for Cooper pairs). The crystallization can now occur at low triplet concentrations because the effective size of the triplet pair (composite particle) can be large.

The effect of pressure on the dimer singlet interactions in SCBO straightforwardly tunes the ground state at *H=*0, consonant with the original Shastry–Sutherland picture. More surprisingly, the crystallization of spin-triplet bosons at high magnetic field also appears to be highly malleable. As applied, pressure increases *x ≡ J′/J* and drives the dimers into the quantum-phase transition, it increases the single–triplet kinetic energy, and thereby increases the linear size *l*_B_ of *S*_*z*_=2 triplet pair bound states. The crystallization of triplet bound states explains the emergence of low-magnetization plateaus, such as *m*≤1/8, for which the average inter-triplet distance *d*_a_=*m*^−1/2^ is significantly bigger than the range of the repulsive bare interactions. Triplet bound states start ‘seeing each other' when *l*_B_ becomes comparable to *d*_a_. The increase of *l*_B_ with pressure could explain the new pressure-induced low-magnetization plateaus (*m*<1/8) that we are reporting here. In this picture, the pairs of triplet bound states are forming larger composite particles under pressure and thus interact at lower *m* concentrations.

## Methods

### Single-crystal growth

Single-crystal samples of SrCu_2_(^11^BO_3_)_2_ were cut from the same high-quality parent single crystal employed in previous experiments^15^,^18^,^20^,^24^,^26^, grown in a floating zone image furnace at a rate of 0.2 mm h^−1^ in an O_2_ atmosphere^35^.

### High-pressure magnetization

The sample's magnetization as a function of field, temperature and pressure was determined in a 35 T resistive magnet at the National High Magnetic Field Laboratory using a ^3^He cryostat with a base temperature of 350 mK and a piston cylinder cell (PCC) (Supplementary Fig. 1). The cell body was made from MP35N, an alloy with a strength ∼1,800 MPa, considerably larger than that of BeCu (∼1,200 MPa). The sample platform was immersed in a polytetrafluoroethylene (PTFE) cup filled with Daphne 7474 oil as a pressure medium^36^. A transducer was used to accurately monitor the load that was applied to the PCC before tightening the top clamp of the cell. The pressure in the sample chamber was measured using ruby fluorescence^37^ at room temperature and at ∼2 K to account for any variation in pressure during the cooling process. The pressure cell was immersed directly into ^3^He to maximize thermal contact. Temperature was measured using a calibrated Cernox thermometer located ∼2 mm from the cell.

### Tunnel diode oscillator

TDO measurements were conducted on cylinder-shaped crystals with approximate dimensions of 2 mm in length by 0.5 mm in diameter. Each crystal was mounted inside a detection coil placed in the pressure chamber. The coil forms the inductive component of a TDO circuit^38^ tuned to operate at a resonant frequency ranging between 10 and 50 MHz (Supplementary Fig. 2). The coil axis and c - axis of the sample were co-aligned with the axis of the pressure cell, parallel to the applied magnetic field. The TDO technique is often used to measure the surface conductivity of metals for measurements of the Fermi surface^39^, but when used to probe the magnetization of insulating materials, it is sensitive to changes in the magnetic moment ∼10^−12^ e.m.u., allowing sensitive high-field measurements of samples in the tight confines of a pressure cell.

To validate the TDO measurements, magnetic torque measurements were conducted at ambient pressure, using commercial piezoresistive atomic force microscopy (AFM) cantilevers (Seiko PRC400)^40^. A 200 × 200 × 100 μm^3^ SCBO crystal was fixed with silicone grease to the end of the 400-μm long cantilever arm. A piezoresistive element at the opposite end of the cantilever senses the deflection of the arm. An additional piezoresistive element on the same cantilever assembly, along with two adjustable external resistors, forms a Wheatstone bridge that was balanced to provide a null signal at zero magnetic field. Changes in sample magnetization induce a torque on the cantilever, resulting in a voltage across the bridge. Supplementary Fig. 3 compares our TDO (red) and magnetic torque measurements (blue) at ambient pressure. The plateau at ∼27 T in the torque magnetometry measurement agrees well in field scale with the feature seen in the TDO data. This plateau is known to correspond to *m=M(H)*/*M*_sat_=1/8 (refs 16, 17, 18).

### Density matrix renormalization group

To check for finite-size effects, DMRG calculations of normalized magnetization curves were performed on lattices of 8 × 8, 10 × 10 and 16 × 8 spins with cylindrical boundary conditions for *x ≡ J′/J*=0.6 and 0.63, and for *x*=0.7, corresponding to the singlet and plaquette states, respectively. Supplementary Fig. 3 includes the normalized magnetization curve *m=M(H)*/*M*_sat_ calculated for *x*=0.63 using the DMRG method for a 16 × 8 lattice, showing the correspondence in field scale between the calculated *m*=1/8 plateau, and the observed feature in torque magnetometry and TDO measurements on SCBO at ambient pressure.

As shown in Supplementary Fig. 4, the magnetization curves merge as a function of system size, with qualitative differences between the two families of curves for *x*=0.6 and 0.7, as would be expected from the differing ground states. Focusing on the *m*<1/3 regime, large steps are visible in small lattices, indicating that some values of the magnetization are not stable, likely due to incommensuration effects.

We show in Supplementary Fig. 5, the calculated singlet–triplet gap for an 8 × 8 lattice with cylindrical boundary conditions, as a function of *x* and the energy for *S*_*z*_=8 (corresponding to *m*=1/8). Both quantities decrease with increasing *x*, showing that the energy required to form the 1/8 plateau in SCBO decreases as a function of increasing pressure. The geometry used (Supplementary Fig. 5 inset) was found to be less sensitive to boundary effects and is similar to the one employed in previous studies^20^.

The DMRG calculations were run keeping up to 3,000 states, giving an upper bound on the truncation error of order 10^−5^. We plot in Supplementary Fig. 6, the ground-state energy per lattice site (*E/N*) as a function of the number of DMRG states (*m*), calculated for an 8 × 8 lattice with open boundary conditions for *x=*0.7 and *S*_*z*_=6. The results illustrate the energy convergence with increasing number of DMRG states.

### Data availability

The data that support the findings of this study are available from the corresponding authors upon request.

## Additional information

**How to cite this article:** Haravifard, S. *et al.* Crystallization of spin superlattices with pressure and field in the layered magnet SrCu_**2**_(BO_**3**_)_**2**_. *Nat. Commun.* 7:11956 doi: 10.1038/ncomms11956 (2016).

## Supplementary Material

Supplementary Information Supplementary Figures 1-6.
