# Supplementary material for: Crystallization of spin superlattices with pressure and field in the layered magnet SrCu2(BO3)2
Source: Nat Commun. 2016 Jun 20;7:11956. doi: 10.1038/ncomms11956 (PMC4915149; doi:10.1038/ncomms11956)
Supplement: Supplementary Information — Supplementary Figures 1-6. [file ncomms11956-s1.pdf]

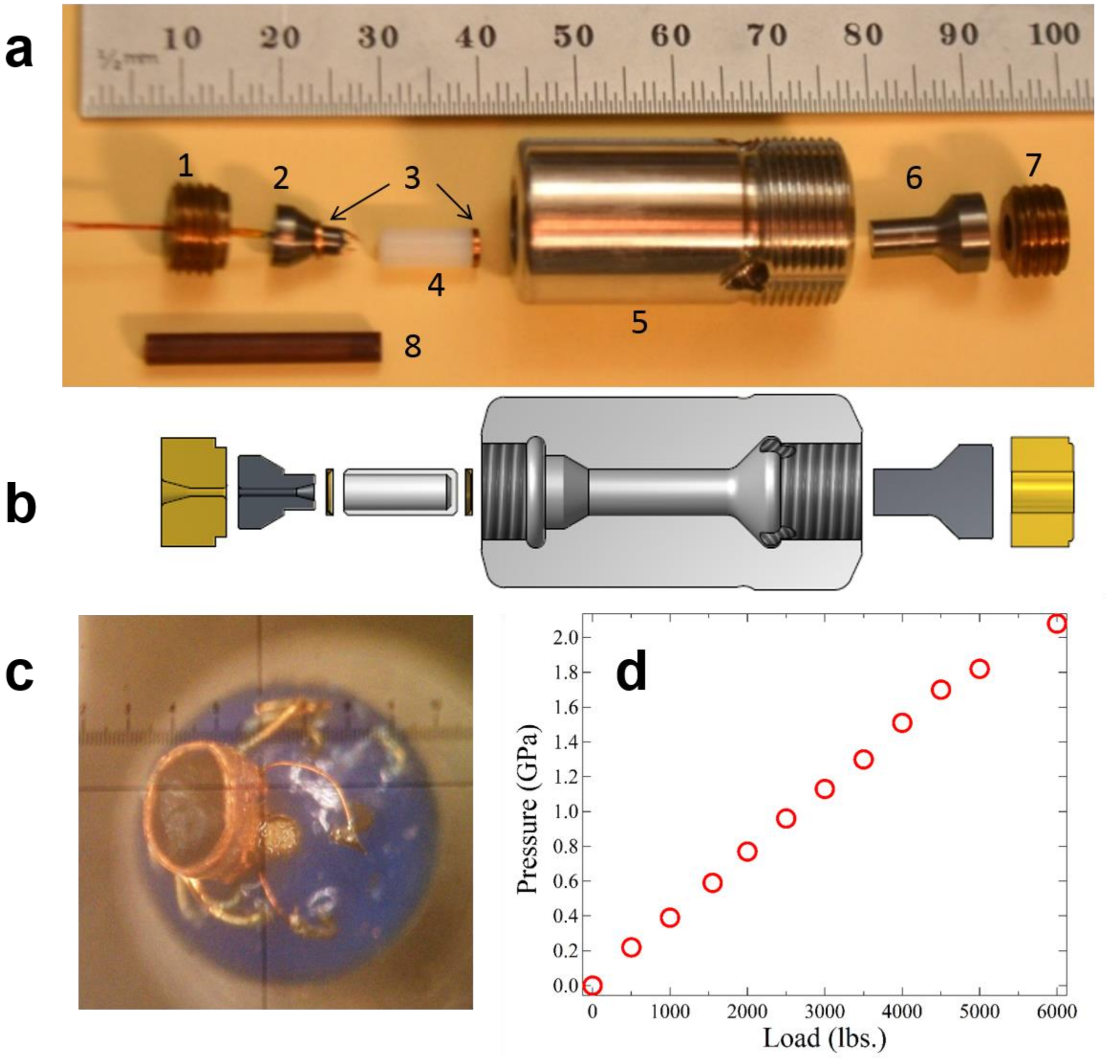


**Supplementary Figure 1 | Piston cylinder cell assembly used in the magnetisation measurements of SBCO**. **(a)** Photograph of the components of the piston cylinder cell: (1) Lower BeCu clamp with thru-hole for wires and optical fibre for pressure measurements. (2) Carbide sample platform with a seat for the lower BeCu obturating ring (3) to seal against the PTFE cup (4). An upper ring (3) prevents the cup from extruding past the WC piston (6) within the bore of the MP35N body (5). An upper BeCu clamp (7) locks in the pressure with a thru-hole for access with a 3.5 mm OD carbide ram. A plastic coupler is used to join 1.0 mm and 0.3 mm OD optical fibres together for the pressure measurement. **(b)** Cross-section illustration of the components. The cell body allows for a maximum sample space compression of 4.0 mm, which yields a pressure exceeding 2 GPa. **(c)** Photo of the top view of a SCBO crystal in a coil attached to the sample platform. The elliptical cross section provides a better filling factor. **(d)** Load

versus pressure curve for the cell.

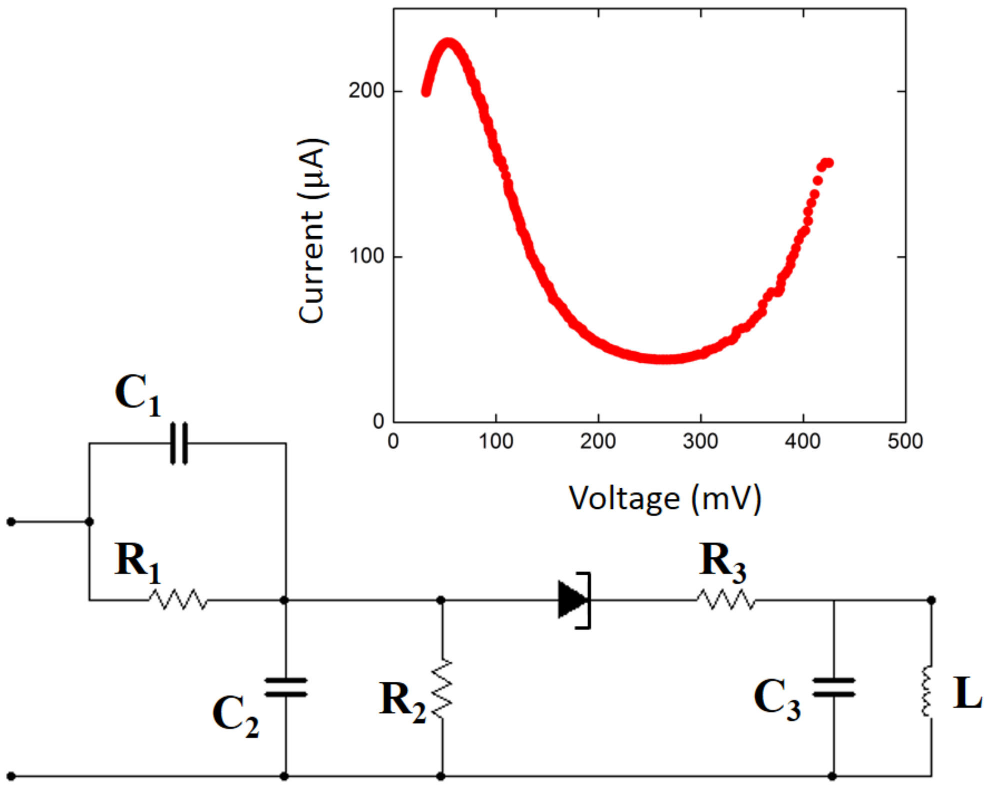


**Supplementary Figure 2 | Schematic of a tunnel diode oscillator (TDO) resonant circuit**. The resistors R1 and R2, act as a voltage divider for controlling the bias voltage of the tunnel diode. C1 isolates the circuit from the room temperature detection electronics and C2 provides a low impedance path from the tank circuit to the diode. When the tunnel diode is biased into the "negative resistance" regime of the I-V curve (top panel), the diode acts with R3 as an *ac* voltage source at the resonant frequency of the tank circuit. The tank capacitor (C3) and the sample coil (L) set that frequency. Changes in the sample's susceptibility alter the value of L and hence the resonant frequency[34].

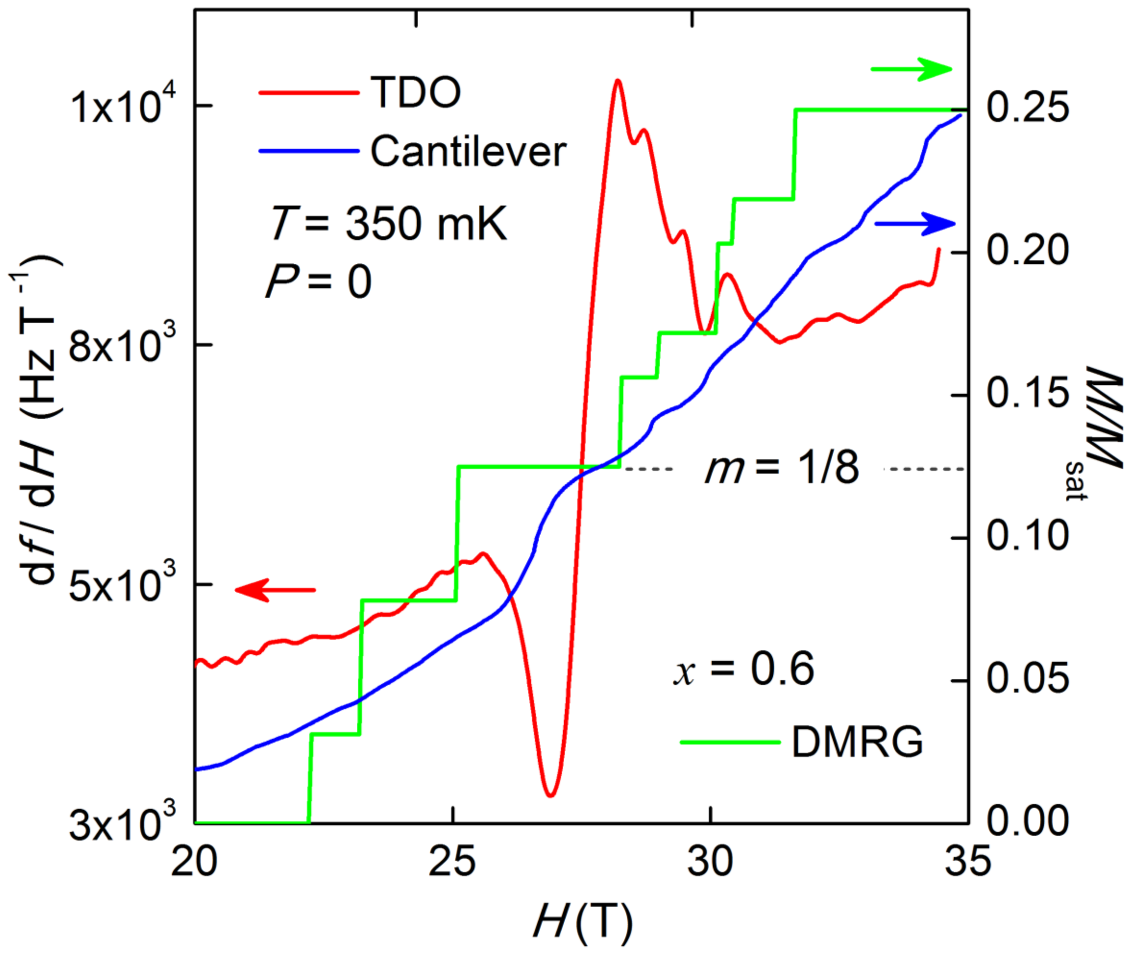


**Supplementary Figure 3 | Comparison between TDO and torque magnetometry measurements and DMRG calculations**. TDO (red) and cantilever torque magnetometry (blue) measurements collected at $T$ = 350 mK and $P$ = 0, and DMRG results calculated for $x$ = 0.6 in a 16×8 spin lattice with cylindrical boundary conditions (green). The experiments and the calculation both show a feature at 27 T associated with the $m$ = 1/8 magnetisation plateau.

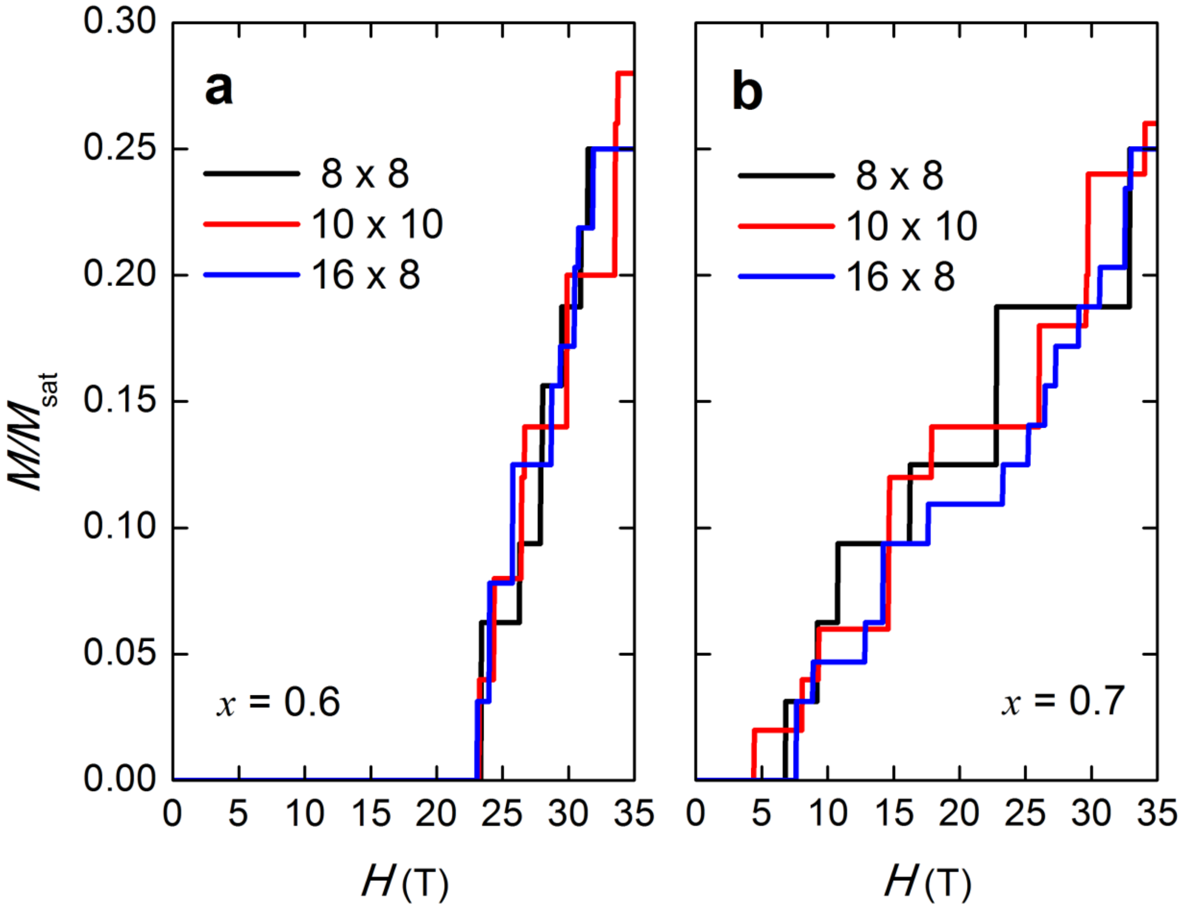


**Supplementary Figure 4 | DMRG calculations of magnetisation curves before and after the quantum phase transition**. **(a)** Magnetisation vs. field for $x = J'/J = 0.6$ (singlet) and **(b)** for $x = 0.7$ (plaquette) as a function of spin lattice size, calculated with cylindrical boundary conditions. The results merge as a function of lattice size and give qualitatively different results for $x = 0.6$ and 0.7.

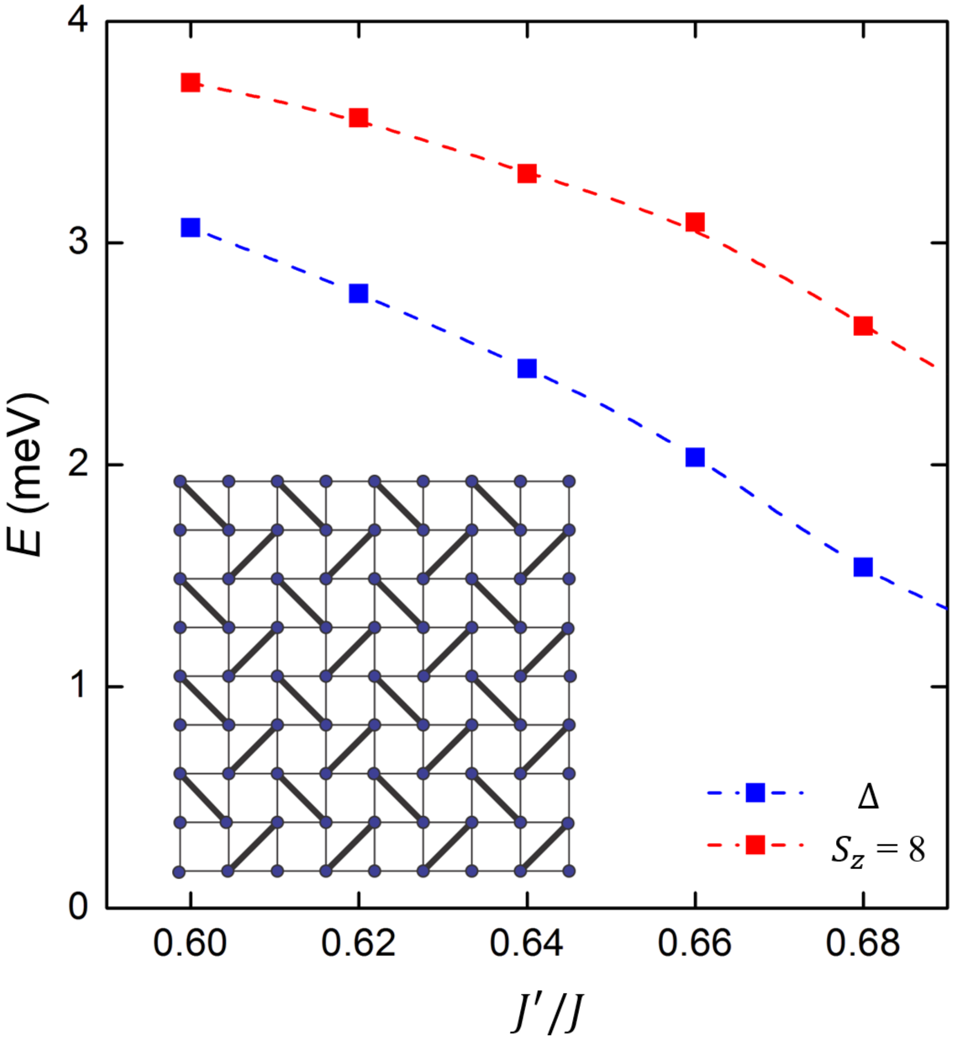


**Supplementary Figure 5 | Calculated singlet-triplet gap and *Sz = 8* energies.** Energy calculated as a function of $x$ for the singlet-triplet gap (Δ) at zero field, and for $S_z$ = 8 (corresponding to $m$ = 1/8) in an 8×8 lattice with cylindrical boundary conditions. The numerical errors are of the order of the symbol size. **Inset:** Lattice geometry, with cylindrical boundary conditions, used for the DMRG calculations.

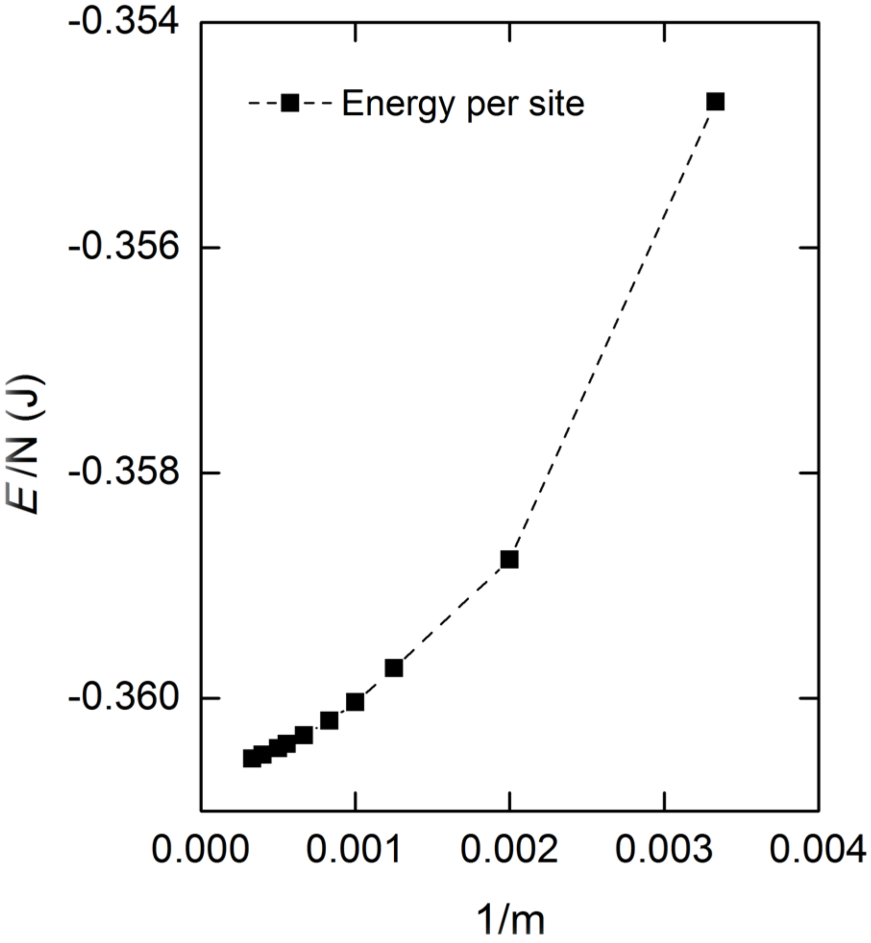


**Supplementary Figure 6 | Energy convergence with increasing number of DMRG states.** The ground state energy for an 8×8 lattice with open boundary conditions, as a function of the number of DMRG states (m), for $x = 0.7$ and $S_z = 6$. Energy is in units of $J$.
